# Influence of Extended Photoperiod Using Blue Light Masks on Hypertrichosis, Coat Condition and General Health Parameters in Horses with Pituitary Pars Intermedia Dysfunction †

**DOI:** 10.3390/ani15192905

**Published:** 2025-10-05

**Authors:** Sinead Parmantier, Panoraia Kyriazopoulou, Margaret McClendon, Amanda Adams, Barbara A. Murphy

**Affiliations:** 1 School of Agriculture and Food Science, University College Dublin, Belfield, Dublin 4, Ireland; sineadcoleman7@gmail.com; 2 Equilume Ltd., Ladytown Business Park, Naas, W91 TP22 Co. Kildare, Ireland; rhea.kyriaz@gmail.com; 3 M.H. Gluck Equine Research Center, Department of Veterinary Science, University of Kentucky, Lexington, KY 40546, USA; maggie.greiter@uky.edu (M.M.); amanda.adams@uky.edu (A.A.)

**Keywords:** PPID, blue light, hypertrichosis, clinical signs, hair coat, horses, health

## Abstract

Fifty-two horses aged >15 years, diagnosed with pituitary pars intermedia dysfunction (PPID), and displaying hypertrichosis were recruited via an online survey of PPID horse owners. From mid-December, group T (*n* = 29) wore Equilume^®^ light masks extending photoperiod to 15 h daily, while group C1 (*n* = 23) remained under natural photoperiod. As 85% (44/52) of recruited study horses received pergolide medication, a second unmedicated PPID research herd (C2; *n* = 17) was recruited and remained under natural photoperiod. Hair coat samples, shedding and body condition scores were collected monthly by owners for 13 months and analysed by the research team. Data related to management, coat condition and PPID clinical signs were collected using bimonthly questionnaires (BMQ). Time (*p* < 0.001), group (*p* = 0.025) and time-by-group interaction (*p* = 0.005) affected hair length. Group differences were attributable to shorter hair lengths in C2, and no differences in hair length occurred between T and C1 (*p* > 0.05). Time affected shedding scores (*p* < 0.001) which was advanced by one month in T (*p* < 0.05). In group T, BMQ responses showed improved coat condition in April (*p* = 0.035), decreased fat coverage in April and June (*p* < 0.05), and increased energy/alertness in February (*p* = 0.022). Improvements in coat condition (*p* = 0.043), clinical signs of PPID (*p* = 0.018), and general quality of life (*p* = 0.035) were reported in T only in a final study questionnaire. Blue light treatment merits further investigation as a complementary treatment for PPID.

## Simple Summary

This study involved 52 owner-recruited horses over 15 years old with pituitary pars intermedia dysfunction (PPID), a condition causing excessive hair growth. The horses were divided into two groups. One group (29 horses) wore blue light masks from mid-December to extend their daylight exposure to 15 h daily. The other group (23 horses) acted as a control and experienced the natural changes in the light/dark cycle. As most (44/52) of these horses received daily medication to treat PPID, a second control group of unmedicated research horses (17 horses) was also recruited and experienced natural daylength changes for 13 months. Researchers measured hair length, hair shedding, and body condition monthly and collected owner feedback about other symptoms every two months. There was no difference in hair lengths between the blue-light-treated and the first control group, but the second control group had shorter hairs. Shedding started a month earlier in the spring for the blue light group. Owners reported better coat condition in April, less fat coverage in April and June, and more energy in February for the blue light group. A final survey showed improved coat condition, fewer PPID symptoms, and better quality of life in the blue light group only. These findings suggest that blue light masks can help manage PPID symptoms and warrant more research.

## 1. Introduction

Pituitary pars intermedia dysfunction (PPID), previously known as equine Cushing’s disease, is an incurable degenerative disease with significant health and welfare implications that affects 21% of horses and ponies over 15 years of age [1,2]. The disease is associated with loss of dopamine-producing neurons leading to hyperplasia of the cells of the pars intermedia of the pituitary gland, which causes excessive release of adrenocorticotropic hormone (ACTH), a pro-opiomelanocortin (POMC)-derived hormone, and other pars intermedia products into the bloodstream [1,3].

As ACTH levels fluctuate naturally throughout the year, with a notable spike in autumn [4,5], testing plasma ACTH levels against seasonally adjusted base values is the most common method of veterinary diagnosis [6,7]. The thyrotropin releasing hormone (TRH) stimulation test works by quantifying the stimulatory effect of TRH on ACTH, and is considered more sensitive when seeking to avoid misinterpretation of results in early disease [3]. Recognition of the two most common clinical signs, hypertrichosis and poor coat shedding, is considered pathognomonic for PPID [2,8,9]. Hypertrichosis is the term used to describe the growth of an excessively long coat, that may be localized or generalized, and that fails to shed appropriately [10]. Out of 14 studies published from 1990 to 2016, 11 reported hypertrichosis in over 50% of PPID cases [1]. In horses with PPID, the winter coat often persists into spring and summer due to poor shedding, the cause of which is unknown but has been postulated to be due to an increased secretion of melanocyte stimulating hormone (MSH), or pressure of the enlarged pars intermedia on thermoregulatory areas of the hypothalamus [10]. A longer coat can cause management challenges, especially in warm climates, as it makes temperature regulation difficult, resulting in heavy sweating that can lead to skin abnormalities.

Muscle wastage, suspensory ligament breakdown, tendonitis, desmitis, abnormal sweating, drinking and urination, as well as recurrent secondary infections, are other common clinical signs [1,11,12,13]. Horses with PPID also commonly present as depressed or overly docile [1,14]. Laminitis is a common comorbidity, often reported by owners before other symptoms are observed and before PPID is diagnosed [1]. To date, no single scientific explanation exists for the wide range of clinical signs described, and further research is required to better understand why different combinations of clinical signs occur [3,7]. Age appears to be the only risk factor associated with PPID [2] and all breeds are affected.

Normally, a horse’s coat grows and sheds in response to changes in day length and temperature throughout the year [15,16,17] and is primarily regulated by seasonally fluctuating levels of prolactin [18,19,20]. Specialised retinal receptors in the eye capture short-wavelength blue light [21,22] which is transmitted to the brain to regulate an animal’s circadian and circannual physiology [23]. A primary mediator of this signal is the daily change in melatonin production by the pineal gland [24]. Melatonin and dopamine both inhibit prolactin [25,26] which occurs in a seasonal manner in horses [24]. Receptors in hair follicle cells allow detection of changes in prolactin signalling, influencing the moulting process [27,28]. Temperature also plays an important role in the hair growth cycle [15]. Lower temperatures (below 15 degrees) stimulate growth of a longer hair coat [17] and has been correlated with decreased prolactin concentrations [15].

Currently, pergolide mesylate is the only licenced drug to treat PPID. Treatment is aimed at controlling the clinical signs rather than curing the underlying disease, and lifelong therapy is required. As a dopamine agonist, it compensates for the loss of dopamine-producing neurons by interacting with dopamine receptors. According to a 2020 review, pergolide appears to be successful in improving some clinical signs, such as hair coat quality and laminitis [29]. Despite these lessening of symptoms, many horses continue to suffer from inappetence or anorexia [2,30,31,32,33,34,35,36] as well as depressed attitude and lethargy [2,29,37,38] and these are also reported adverse effects of the medication [8]. Dosing also poses an issue for owners [39], due in part to the low palatability of the pills to horses, such that alternative or complementary treatment options are desirable and warrant exploration.

The use of artificial blue light has been shown to regulate seasonal reproductive physiology and hair coat changes in horses [20,40,41,42,43]. A recent preliminary study using blue light masks to extend daily photoperiod was shown to reduce hair growth in horses with PPID [44]. When daylength was maintained at 15 h per day from mid-July, hair weights were found to be lower in late October than in a control group maintained under natural photoperiod [44]. Additionally, a study in healthy horses and ponies showed that blue light treatment could maintain a shortened summer coat when initiated in mid-summer and accelerate shedding of the winter coat when initiated in mid-winter [20]. Together, these studies suggest that using blue light may have potential as a non-invasive management tool for mitigating hypertrichosis. Furthermore, daytime exposure to blue light optimally entrains the circadian system and promotes strong circadian synchrony, which has been associated with improved muscle development in horses [45] and strengthened immunity, improved cognition and alertness, and reduced overall health issues in animals and humans [23,46,47,48,49,50]. The current study aimed to further investigate the influence of extended photoperiod using blue light on hypertrichosis, coat condition and general health parameters in horses diagnosed with PPID [51].

## 2. Materials and Methods

### 2.1. Study Population Selection

#### 2.1.1. Recruitment Questionnaire

To identify suitable study participants, a Qualtrics^®^ questionnaire was designed for circulation to owners of PPID horses within the northern hemisphere. The first section of the questionnaire asked a series of yes/no questions to determine eligibility for inclusion in the study. Eligibility criteria were set as horses aged >15 years, with a veterinary diagnosis of PPID, not having commenced medication to treat PPID within 6 months of the study start date, and displaying hypertrichosis. Pregnant mares, mares involved in a breeding programme, horses that had their hair coat fully clipped within 2 months of the study start date, and horses who had started medication for PPID or had been diagnosed with PPID within 6 months of the study start date, were deemed ineligible. Respondents answering ‘Yes’ to a question on the use of artificial lighting to influence seasonal hair coat growth were also excluded. Respondents were asked to confirm that they could limit their horse’s exposure to artificial lighting after 6 pm for the duration of a 13-month study. Respondents had to agree to the study conditions, consent to be contacted by researchers and have their personal information stored for the study period.

The second section of the questionnaire comprised a set of questions related to horse demographics and management conditions. In the final section, respondents completed a multiple choice question indicating the common clinical signs of PPID and co-morbidities that their horse displayed, as outlined by the EEG [8]. To simplify options, clinical signs, such as loss of topline and muscle atrophy were merged, while others such as delayed shedding, loss of seasonal hair shedding, patchy hair growth, regional hypertrichosis and general hypertrichosis remained as discrete options, in line with the focus of the study (see Supplementary Materials for questionnaire).

#### 2.1.2. Questionnaire Distribution

Summary details of the study and an invitation to participate, along with a link to the questionnaire, were circulated to the Equine PPID (Cushing’s) Facebook group (28.1 K members) and the Equine Cushing’s disease support group for owners Facebook group (circa 8 K members) and were also posted to the websites and social networks of University College Dublin, and the University of Kentucky. It was also shared online via the Equilume Facebook page, Equine Science Update, Sidelines magazine, and the Irish Field news publication. Potential participants were informed that they would be assigned to either a treatment or control group, but that all participants would receive a light mask (Equilume^®^), either at the beginning of the study (treatment group) or at completion of the study (control group). The questionnaire was open to responses from 1 October 2021 to 23 November 2021.

#### 2.1.3. Screening of Potential Participants

**Figure 1:**
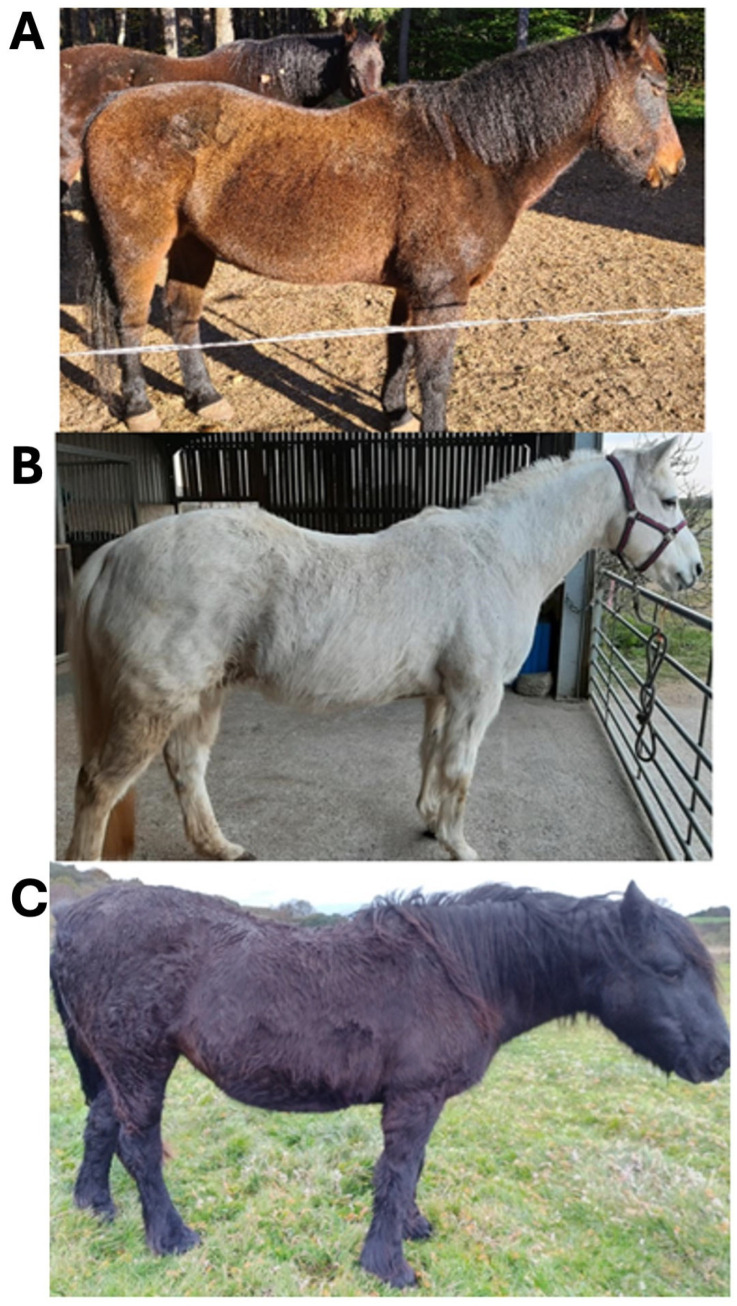
Photographic examples and description of each hypertrichosis score (1–3) assigned to study horses. (**A**) 1: Regional hair coat abnormalities/long hair growth restricted to discrete areas; (**B**) 2: Generalised hair coat abnormalities, slightly to moderately long hair coat; and (**C**) 3: Severely long and/or curly hair coat over the entire body.

Of 501 fully completed questionnaires, 113 potential participants met the criteria for inclusion in the study. Each was then requested to provide photographic evidence of their horse or pony displaying hypertrichosis and written evidence of a diagnosis of PPID. For the latter, a signed letter from a veterinarian or a copy of endocrine test results confirming PPID, in accordance with the reference ranges provided by the Equine Endocrine Group [14], were deemed acceptable. Where ACTH levels or post-TRH ACTH levels exceeded the reference ranges listed in a horse’s bloodwork documents, these results were also deemed valid. Failure to provide the requested documents confirming PPID or commencing pergolide medication within 6 months of the study start date eliminated a further 61 respondents, leaving 52 final study participants. Each participant’s horse was assigned a unique ID number, used thereafter to refer to that animal’s samples or the owner’s questionnaire responses. Each participant’s horse received a baseline hypertrichosis score by scoring the photos received on a scale of 1–3, as follows: 1 = regional hair coat abnormalities i.e., long hair growth restricted to discrete areas (lower jaw, jugular area, and palmar or plantar aspects of limbs); 2 = generalised hair coat abnormalities, slight to moderate long hair coat; and 3 = severely long and ⁄or curly hair coat over the entire body [9]. Representative horses for each score are presented in Figure 1.

#### 2.1.4. Animals

The mean age of participant horses was 23.4 (±4.0), with a range from 17 to 33 years, and comprised geldings (*n* = 28) and non-pregnant mares (*n* = 24). All breeds were accepted, but breed ‘type’ was defined by researchers as either cold blood type (native breeds and draught breeds) or ‘Other’ (warmblood and hot blood breeds) as determined by size, physical features (presence or absence of feathering), and owner descriptions. Participant horses included both those who were medicated (*n* = 44) and those who were unmedicated (*n* = 8) for PPID with pergolide mesylate. All study horses were based in northern hemisphere countries.

#### 2.1.5. Experimental Design

Horses were blocked by sex, breed type and hypertrichosis score and assigned to treatment (T) and control (C1) groups. As 85% (44/52) of recruited participant horses received long-term pergolide treatment, a second unmedicated control group (group C2) was recruited from the University of Kentucky’s PPID research herd to inform on seasonal changes in hair length in a group of unmedicated and untreated horses. Although horses in group C2 met all of the study criteria, they were managed differently to the other groups, living together outdoors as a herd at the same location and consisting of the same breed type (Other). A complete breakdown and demographics of the experimental groups is described in Table 1.

#### 2.1.6. Blue Light Therapy

Blue light masks (Cashel Light Mask, Equilume Ltd., Naas, County Kildare, Ireland) were posted to participants in group T prior to the beginning of the study. Once fitted, the light mask directed low-intensity (50 lux) blue light (468 nm) to the right eye. Once activated, the blue light turned on automatically each day at 08:00 h and turned off at 23:00 h, consistently extending day length to 15 h. Participants in group T were advised to fit the masks on their horses on 15 December 2021. Instructions on how to activate, fit and charge the masks weekly were provided by email to all participants by way of ‘how to’ videos. Participants were instructed to leave the mask on their horse for the duration of the study period, only removing it to groom, exercise, or to charge the mask. Control animals in group C1 and group C2 remained exposed to natural photoperiod.

### 2.2. Sample and Data Collection

Prior to the commencement of the study, all participants received a study kit containing 13 envelopes labelled for each month of the study. These contained scorecards for the collection of body condition scores (BCS) and shedding scores, small plastic hair sample collection bags, a printed guide for the Henneke body condition scoring system [52] and pre-addressed return shipping envelopes.

Beginning 15 December 2021, and continuing monthly thereafter until 21 December 2022, participants collected a hair sample, and recorded a hair shedding score, and a BCS. Instructional ‘how to’ videos were provided for each of these. For hair samples, participants were instructed to pluck a minimum of 20 full-length hairs (ensuring hair follicles were attached) from within a 10 × 10 cm area below the withers. To calculate shedding scores, participants were requested to run their hand along the horse’s back three times, from withers to croup, and observe the number of hairs coming away on their hand, grading the number as follows: 1 = no hairs, 2 = a few hairs, 3 = many hairs, 4 = many hairs and hairs falling to the ground. For reporting BCS using the Henneke body condition scoring system, participants were asked to observe and palpate 5 key areas: the crest of the neck, the withers, behind the shoulder, along the ribs, and down along the tail-head, assigning a score to each area, the average of which was recorded on the scorecard provided. Samples and scorecards were shipped to the research team every 3 months.

#### 2.2.1. Processing of Hair Samples

Sample processing commenced on receipt of each three-month sample batch. Prior to being measured, unprocessed samples were stored in their collection bags and envelopes in a dry, secure location. Using the unique participant ID as an identifier, 10 full-length hairs with visible hair follicles were selected from each monthly sample and laid flat on a brightly coloured card to aid with visibility. The longest guard hairs (longer and thicker than under hairs) were selected from each sample. Clear tape was used to adhere the straightened hairs to the card and each hair measured to the nearest mm. The mean hair length was calculated and recorded.

#### 2.2.2. Bi-Monthly Questionnaires (BMQs)

A bi-monthly questionnaire (BMQ) was designed using Qualtrics and sent to participants for completion in February, April, June, August, October and December 2022. Questionnaires were pre-tested by a group of horse owners (*n* = 5) and edited for clarity based on feedback. Participants responded to the same set of questions in each BMQ but were asked to limit their responses to information pertaining to the previous two-month period only.

The BMQ collected information on medication status, health indicators (coat condition, energy and alertness, fat coverage, hair length, hair shedding, body condition, muscle tone, appetite, sociability, sweating, drinking, urination and stereotypical behaviours), the occurrence of common negative health incidences associated with PPID (sole abscesses, respiratory infections, skin infections and corneal ulcerations [1]), dry eye and stomach ulcers [53,54], and comorbidities as outlined by the EEG [8]. Participants were asked yes/no type questions about whether they had observed any change in each parameter in their horse over the previous two months. If they answered ‘No,’ this was recorded as ‘No change.’ If they answered ‘Yes,’ they then had to indicate the direction of change (increase/improvement or decrease/disimprovement). Participants were asked to provide any additional information in an open text box. The final section of the BMQ aimed to capture management changes related to housing, blanketing, artificial light exposure, feed or feeding routine, and the provision of nutritional supplements.

#### 2.2.3. Final Questionnaire

A final end-of-study Qualtrics^®^ questionnaire was circulated to participants at the end of the study period. Participants were asked to indicate whether their horse’s PPID clinical signs had increased, decreased or not changed over the entire 13-month period of the study. If a change in clinical signs was indicated, participants then selected the specific clinical signs that had increased and decreased from a provided list of PPID clinical signs (as outlined by the EEG [8]). Participants were also asked to indicate whether they felt their horse’s overall quality of life (QoL) and coat condition had increased/improved, decreased/disimproved or not changed during the study period.

#### 2.2.4. Environmental Temperature Data

Online weather data for the location closest to each participants’ horse was used to compile the average monthly temperatures in December 2021 and throughout 2022 in each group. Temperatures of United States locations were gathered using the National Oceanic and Atmospheric Administration, National weather service (https://www.weather.gov, accessed on 1 March 2023). For all other locations, monthly averages calculated from several years were used and were compiled using Weather Spark© (https://weatherspark.com, accessed on 1 March 2023).

### 2.3. Data Analysis

All statistical tests were performed using GraphPad Prism version 10.0.2, except for Fisher’s Exact tests, which were performed using IBM SPSS statistics version 27. Significance for all tests was set at *p* < 0.05.

#### 2.3.1. Group Statistics

*t*-tests were applied to assess differences in mean age and location latitude between groups T and C1. Chi-squared or Fisher’s exact tests were applied where appropriate to assess differences between groups for sex, breed ‘type,’ exercise status and hypertrichosis score (1–3).

#### 2.3.2. Hair Length

Multiple imputations were used to estimate missing hair length values where three or fewer hair lengths were missing from a single participant’s horse. The average of the previous and subsequent length to the missing value was imputed. This imputation was used for five missing values across the study cohort. Multiple imputations were not deemed appropriate when more than three values were missing from a single horse, and the horse was removed from this analysis where this occurred. Linear mixed models explored the effect of group (T, C1, C2), time, and group x time interactions on hair length, followed by Tukey’s or Sidak’s multiple comparisons tests where appropriate. Individual horse ID was set as a random effect. Model assumptions and normality were checked using histograms, Q–Q plots and Shapiro–Wilks tests.

#### 2.3.3. Hair Shedding Scores and BCS

Multiple imputations were used to compute missing scores where three or fewer scores were missing. This imputation was used for 8 missing values. Hair shedding scores and BCS were analysed as ranked data using Friedman tests to assess within-group variation, followed by Mann–Whitney tests to check for differences between groups at specific time points.

#### 2.3.4. BMQs

The categorical responses for each health indicator were as follows: Increased/improved, decreased/disimproved, and no change. The categorical responses regarding blanketing were condensed into two outcomes: ‘Blanketed (‘all day’ and/or ‘all night’ and/or ‘at pasture’)’ and ‘Never blanketed.’ Associations between groups and response frequencies in relation to each health indicator were assessed using Fisher’s exact tests. The cumulative presence or absence of negative health incidents and comorbidities between groups was examined using Fisher’s exact tests.

#### 2.3.5. Final Questionnaire

The categorical responses for changes in health and behaviour parameters that the participants reported over the 13 months of the study were as follows: Increased/improved, decreased/disimproved, and no change. Response frequencies for hypertrichosis related clinical signs (regional hypertrichosis/hypertrichosis/patchy hair growth/delayed hair coat shedding/loss of seasonal shedding) were summed and assessed separately. Fisher’s exact tests were used to determine associations between groups.

## 3. Results

### 3.1. Group Statistics

No differences in age, sex, or hypertrichosis score were observed between groups (*p* > 0.05). Groups T and C1 did not differ for proportions of breed ‘type’ or mean latitude (*p* > 0.05). Group C2 differed from groups T and C1 for these parameters (*p* < 0.05, respectively) in that they were located at the same latitude, which was lower than the mean latitude of groups T and C1, and in so far as they were all the breed type ‘Other.’ The proportion of horses exercised was not different between groups T and C1 (*p* = 0.4), though group C2 horses were not exercised. The proportions of horses with each hypertrichosis score (1–3) assigned by researchers were similar among all three groups (*p* = 0.44). Detailed information on group demographics is presented in Table 1.

Horses in group T and group C1 were based in different locations in northern hemisphere countries, distributed as follows: France (*n* = 1), Belgium (*n* = 1), Austria (*n* = 1), Norway (*n* = 1), Finland (*n* = 1), Canada (*n* = 4), Germany (*n* = 7), Ireland (*n* = 7), the United Kingdom (*n* = 15), and the U.S.A (*n* = 14). Group C2 horses were all at the same location in the U.S.A (*n* = 17). Among the groups, all but four horses experienced at least one other clinical sign of PPID, in addition to hypertrichosis.

### 3.2. Hair Length

A total of 736 hair samples were received and analysed. Missing samples were due to participant non-compliance, shipping-related issues, euthanasia of horses due to age-related ill-health, or non-viable samples where hairs were broken. Two participants from each group (T, C1 and C2) were removed for the above reasons or if they had not provided a baseline sample (December 2021) or had only provided one sample throughout the study. Final group sizes for hair analysis were as follows: T (*n* = 27), C1 (*n* = 21), C2 (*n* = 15).

**Figure 2:**
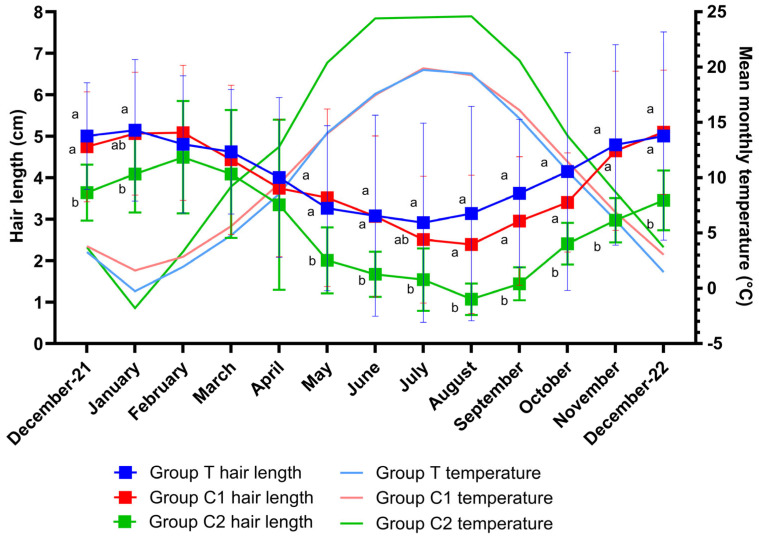
Hair lengths (mean± SD) and corresponding mean monthly temperatures throughout the study for groups T (blue light treatment), C1 (medicated control) and C2 (unmedicated control). There was an effect of time (*p* < 0.001), group (*p* = 0.02) and group by time interaction (*p* = 0.005) for hair length. Differences in hair length between groups for each month are indicated by different letters (a,b represents significance at *p* < 0.05). A significant effect of time was observed for temperature (*p* < 0.0001), but no group (*p* = 0.59) or group by time interaction effect (*p* = 0.53) was observed. There were no significant temperature differences between group means for any month (*p* > 0.05 for all).

Shapiro–Wilks tests confirmed the normality of the data for all groups. There was an overall effect of time (*p* < 0.001), group (*p* = 0.02) and group by time interaction (*p* = 0.005) for hair length. Multiple comparison tests revealed hair length differences (*p* < 0.05) between groups T and C2 for all months except February, March, and April (*p* > 0.05) and differences between groups C1 and C2 (*p* < 0.05) for all months except January, February, March, April and July (*p* > 0.05). Changes in hair length over the study period are graphically presented as means ± SD per month for groups T, C1 and C2 (Figure 2). As differences between groups were only attributable to the significantly shorter hair lengths of group C2, this group was removed from a further analysis such that differences between groups T and C1 could be assessed. A significant effect of time (*p* < 0.001) but no effect of group (*p* = 0.74) or group by time interaction (*p* = 0.24) was observed for hair length between groups T and C1. Multiple comparison tests revealed no differences between group means on any month (*p* > 0.05 for all).

### 3.3. Environmental Temperature

Data were normally distributed and model assumptions were met. There was a significant effect of time (*p* < 0.001), but no group (*p* = 0.59) or group by time interaction effect (*p* = 0.53) for temperature. Multiple comparisons revealed no significant differences between group means at any timepoint (*p* > 0.05). Monthly mean temperatures for each group are graphically presented alongside hair length data in Figure 2.

### 3.4. Shedding Scores

A total of 694 scores were included for analysis. Data from participants for which more than three scores were missing were not included in the analysis. Final group numbers were group T (*n* = 22), group C1 (*n* = 19), group C2 (*n* = 13). An total of 151 scores were not available due to participant non-compliance, shipping issues, and euthanasia of horses due to age-related ill-health. A significant effect of time was observed for shedding scores in group T, C1 and C2 (*p* < 0.001, for all).

**Figure 3:**
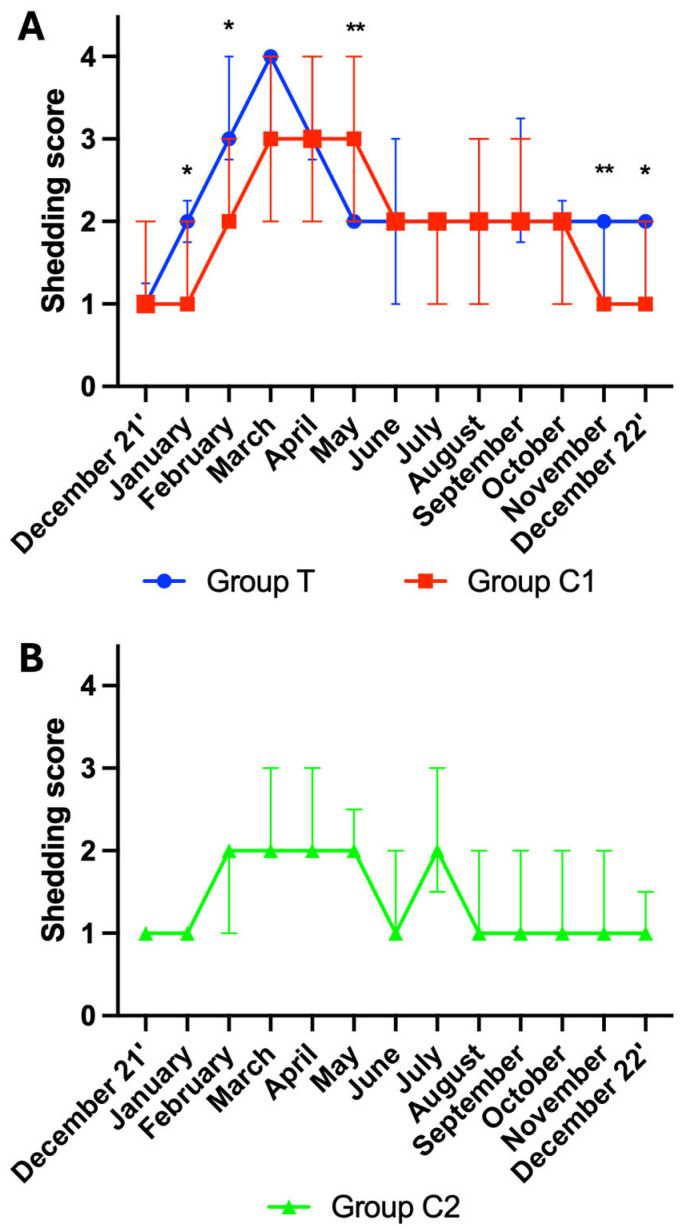
Hair shedding (median ± interquartile ranges) for (**A**) groups T and C1, and (**B**) group C2. T = blue light treatment; C1 = medicated control; C2 = unmedicated control; *, ** indicate significance at *p* < 0.05 and *p* < 0.01, respectively.

Mann–Whitney tests revealed group differences between group T and C1 for the following months: January (*p* = 0.013), February (*p* = 0.016), May (*p* = 0.005), November (*p* = 0.002) and December 22’ (*p* = 0.046). Shedding scores were higher in group T for all but May, where they were lower. As group C2 was distinctly different to group T and group C1, no further multiple comparison analysis was performed on the data from this group. For visualisation purposes, the shedding scores are graphically presented as the median ± interquartile range (IQR) for groups T and C1 (Figure 3A) and for group C2 (Figure 3B).

### 3.5. Body Condition Scores

**Figure 4:**
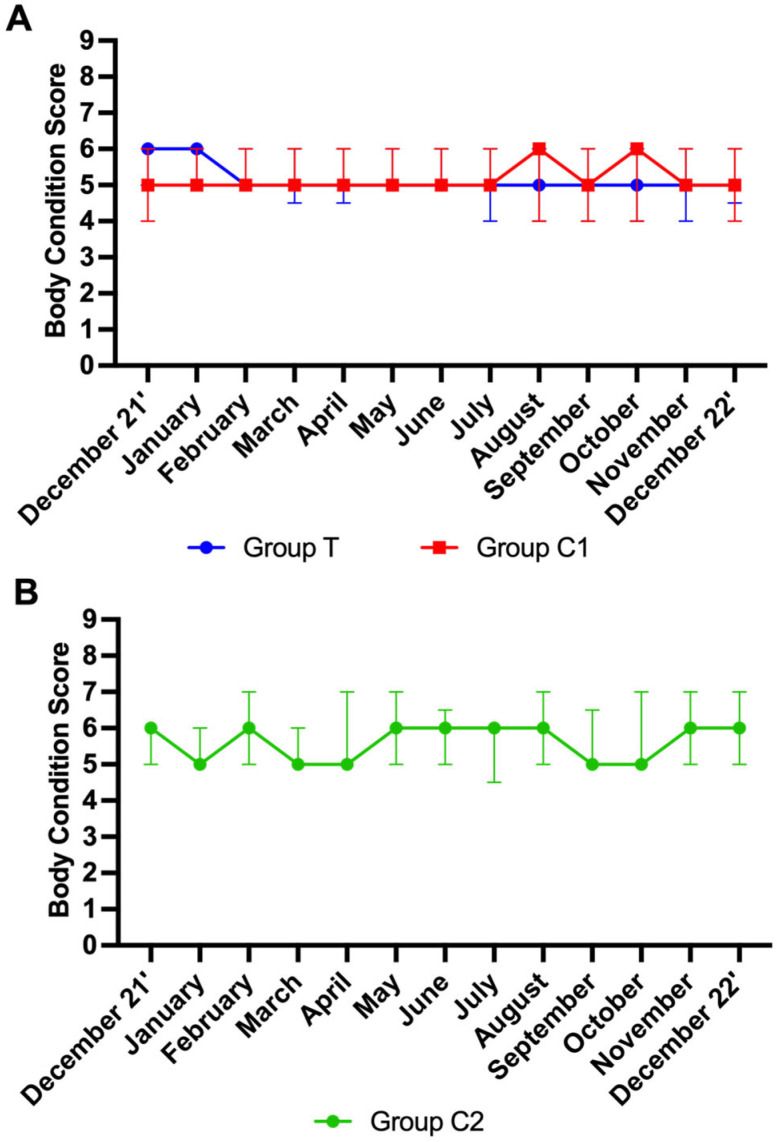
Body condition scores (median ± interquartile ranges) for groups T and C1 (**A**) and C2 (**B**) T = blue light treatment; C1 = medicated control; C2 = unmedicated control.

A total of 681 scores were analysed. Data from participants for which more than three scores were missing were not included in the analysis. Final group numbers were as follows: group T (*n* = 21), group C1 (*n* = 19), group C2 (*n* = 13). Missing scores were due to participant non-compliance, shipping issues, and euthanasia of horses due to age-related ill-health. No effect of time was observed for BCS in groups T and C1 (*p* > 0.05 for both). An effect of time on BCS for C2 was observed (*p* < 0.01). Mann–Whitney tests showed no significant differences between groups T and C1 at any time point (*p* > 0.05). As group C2 was distinctly different to group T and group C1, no further multiple comparisons analysis was performed on the data from this group. For visualisation purposes, BCS are graphically presented as the median ± IQR per month for groups T and C1 (Figure 4A) and group C2 (Figure 4B).

### 3.6. Bi-Monthly Questionnaires (BMQs)

All BMQ data from participants in group T (*n* = 29) and group C1 (*n* = 23) were included in the analysis of BMQ responses. BMQ data were not collected in relation to group C2. The overall response rate was 85% for the six BMQs, 85.4% in group T and 85.5% in group C1.

**Figure 5:**
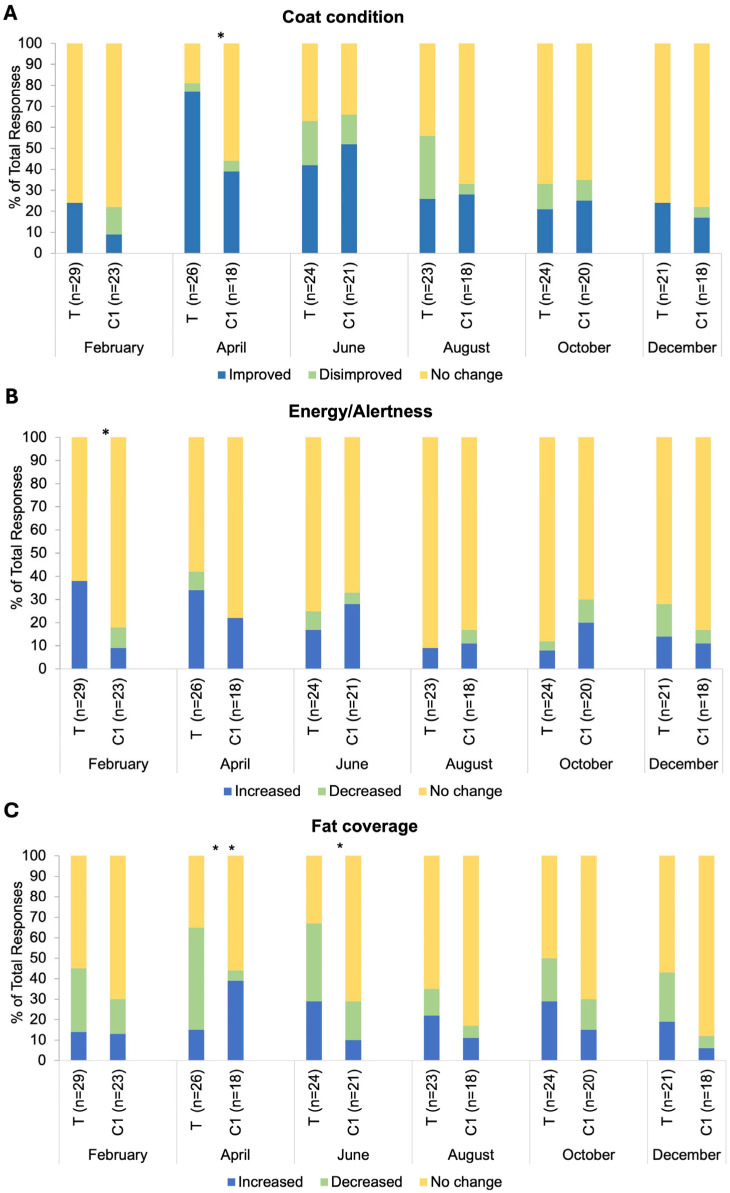
Bi-monthly questionnaire responses for (**A**) coat condition changes, (**B**) energy/alertness, and (**C**) fat coverage, broken down by the following categorical responses: Increased/improved, decreased/disimproved and no change; T = blue light treatment, C1 = unmedicated control. *, * * indicate significance at *p* < 0.05 and *p* < 0.01, respectively.

There was a significant association between group and response frequency in relation to coat condition changes in the April BMQ (*p* = 0.02; Figure 5A). The response ‘Increased/Improved’ was selected by 77% of respondents in group T and 39% in group C1. There was an association between group and response frequency in relation to energy and alertness in the February BMQ (*p* = 0.02; Figure 5B). The response ‘Increased/Improved’ was selected by 38% of respondents in group T versus 9% in group C1. There was an association between group and response frequency in relation to fat coverage for both April and June BMQs (*p* = 0.005, *p* = 0.04 respectively; Figure 5C). The response ‘Decreased/Disimproved’ was reported by 50% of group T respondents versus 5% of C1 respondents in April. No significant differences in the frequency of specific responses between groups for any month (all *p* > 0.05) were observed for changes in medication dosage, other health indicators explored (muscle tone, appetite, sociability, sweating, drinking, urination, stereotypical behaviours) and blanketing. Response frequencies for other negative health incidents (sole abscesses, skin infections, respiratory infections and corneal ulceration, dry eye and stomach ulcers) were very low for the individual incidences reported. Cumulative response frequencies (presence/absence of negative health incidences) did not differ between groups (*p* > 0.05). Comorbidities reported in very low frequencies each month were as follows: insulin dysregulation, laminitis, tendonitis, and desmitis. Cumulative response frequencies (presence/absence of comorbidities) did not differ between groups (*p* > 0.05).

### 3.7. Final Questionnaire

**Figure 6:**
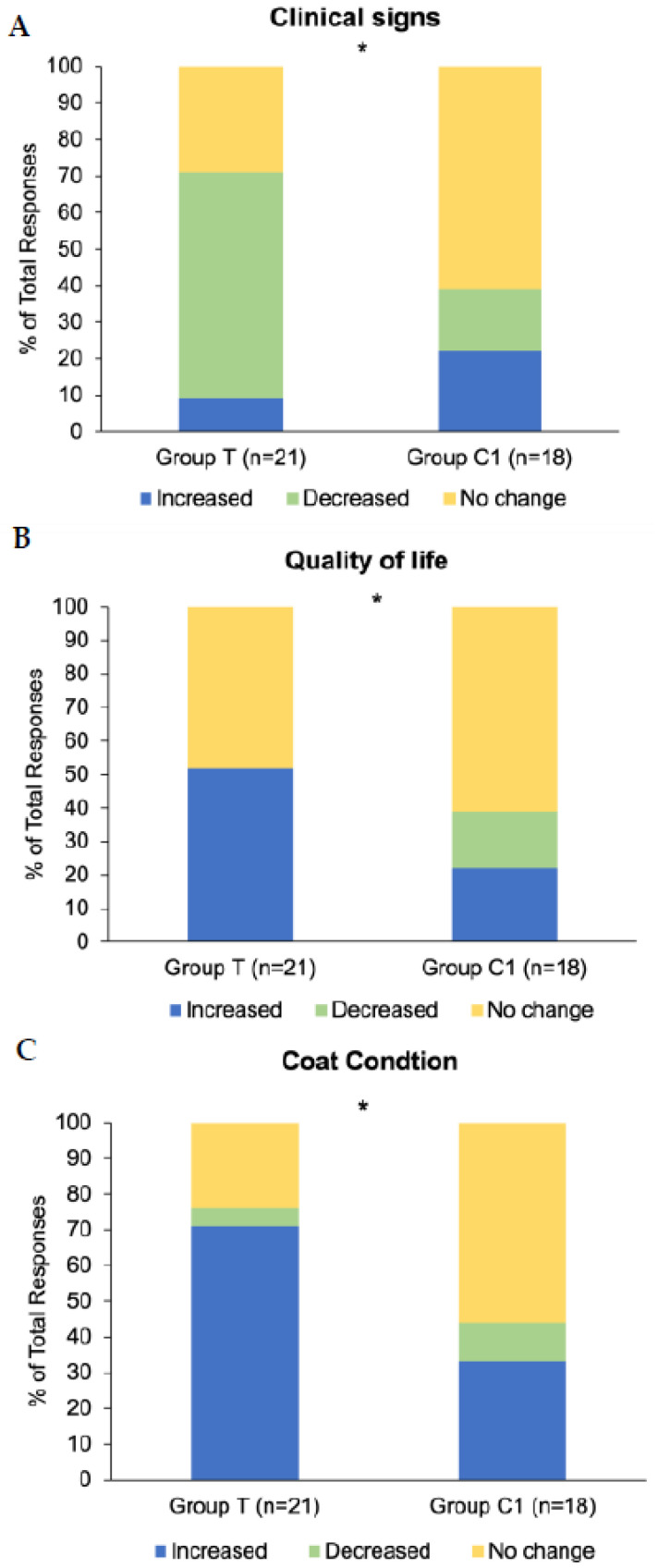
Response frequencies to questions in final study questionnaire related to overall changes in (**A**) clinical signs of PPID, (**B**) quality of life, and (**C**) coat condition. Group T = blue light treatment, group C1 = unmedicated control. * Signifies statistically significant association between group and response (*p* < 0.05).

Twenty-one participants in group T (*n* = 21) and 18 participants in group C1 (*n* = 18) responded to the final questionnaire. Exploration of associations between group (T and C1) and response (increased/improved, decreased/disimproved, and no change) frequency revealed significant associations for changes in clinical signs (*p* = 0.02; Figure 6A), quality of life (*p* = 0.03; Figure 6B), and coat condition (*p* = 0.04; Figure 6C), whereby the response ‘Increased/Improved’ was higher in group T for all three. There were no significant associations between group and response type for questions regarding muscle tone (*p* = 0.31), energy (*p* = 0.15) and sociability (*p* = 0.57). Results for the parameters with significant associations are graphically presented as the percentage of each response as a proportion of the total number of responses to the questionnaire (Figure 6).

#### Reported Changes in Clinical Signs of PPID

A breakdown of each clinical sign and the number of participants reporting changes (increased/decreased) at the end of the study is presented in Table 2. Delayed hair shedding was the clinical sign most reported to have decreased by group T (*n* = 7), followed by regional fat deposits (*n* = 5). In group C1, the most reported decreased clinical sign was laminitis (*n* = 3), followed by regional fat deposits and abnormal sweating (*n* = 2). Muscle atrophy was the most frequent clinical sign marked as ‘Increased,’ by both group T (*n* = 4) and group C1 (*n* = 3).

Cumulative response frequencies (decreased/increased/no change) for hypertrichosis-related clinical signs (regional hypertrichosis/hypertrichosis/patchy hair growth/delayed hair coat shedding/loss of seasonal shedding) were significantly different between groups T and C1 (*p* < 0.001). They were reported as decreased by 71% (15/21) of group T respondents and 6% (1/18) of group C1 respondents, whereas 14% (3/21) of group T respondents and 33% (6/18) C1 respondents reported them as increased.

## 4. Discussion

The aim of this study was to determine if extended daily photoperiod provided by blue light masks affected hypertrichosis, coat condition and general health in horses diagnosed with PPID. Hypertrichosis (the localised or generalised growth of an excessively long coat) and abnormal hair shedding patterns are common clinical signs of PPID, in addition to numerous other conditions resulting from loss of dopamine-producing neurons in the pars intermedia and consequent excessive release of POMC-derived hormones and other products into the bloodstream [2,3].

### 4.1. Hair Length

Hair lengths in the three study groups displayed significant variation over time, reflective of circannual rhythmicity in hair growth cycles in horses and other equids [55]. Synchronous peaks and troughs in hair length were apparent, mirroring a sine wave pattern that is characteristic of a biological rhythm [56], and correlated with times of highest and lowest mean temperatures and daylengths, respectively. As the duration of daily light exposure was fixed at 15 h for the blue-light-treated group, these results suggest that ambient temperature may play a primary role in the regulation of seasonal hair growth. This is supported by a previous study evaluating blue light treatment on pelage in horses and ponies at different times of the year, where the authors concluded that environmental temperature was one of the key entrainment factors for the circannual rhythm in hair coat changes [20]. It was not previously known if PPID impacted the circannual hair coat growth cycle in horses, and the finding of conserved circannual patterns of hair length changes in light-treated, and in both medicated and unmedicated untreated PPID horses confirms that it does not.

Unexpectedly, hair lengths in the blue-light-treated group did not differ from a matched PPID control group (C1) maintained under natural photoperiod conditions. This contrasts with findings in healthy horses that showed a reduction in hair lengths and weights when photoperiod was extended using blue light masks from July until late October [20]. Refractoriness to the long day photoperiod could explain the failure of light treatment to delay the growth of a longer winter coat in the autumn months for group T, as has been observed in studies using light to manipulate reproductive cyclicity in mares [57,58]. However, this cannot explain the failure to observe reduced hair lengths in the spring and summer months in the light treated group.

A recent preliminary study using blue light masks to maintain an extended daily photoperiod in PPID horses reported lower hair weights in blue-light-treated horses compared with controls maintained under natural photoperiod [44]. The study involved horses of a similar breed type known for shorter hair coats (quarter horses and thoroughbreds) and involved weighing entire hairs [20]. The homogeneity of the study population may explain why a difference in hair weight was observed in response to treatment [20]. The treatment groups in the current study were represented by two different breed types, warmblood or hot blood breeds and cold blood breeds. Cold blood breeds have been found to grow longer and heavier hair coats than other breeds [59] and cold bloods represented 31% and 43% of groups T and C1, respectively. Therefore, the accurate measurement of hair weights would have necessitated the trimming of hairs to an equal length prior to weighing to account for greater inter-individual variability in length. This was impractical in the current study. It is possible therefore that hair weights may have differed between the groups. Hair coats respond to increased daylengths and temperatures in spring and summer by producing lighter, finer hairs than those of the thicker winter coat [60]. Indeed, Nolan et al. (2017) observed differences in hair weights between foals born to light-treated versus control mares and these differences exceeded the differences observed in lengths [42], indicating that this may be a more sensitive parameter to evaluate when assessing coat changes in response to light. Thus, these factors may explain the differences in results between analyses of hair weight and hair length in response to blue light therapy in different studies. It is also worth noting for future studies that measurement of hair length may not be a sufficiently informative marker of the degree of hypertrichosis. Hair samples in the current study were collected from a single location beneath the withers and thus may not represent coat changes across the entire body. This may explain why significant differences were observed between groups for coat condition and hair shedding (discussed below), as these changes were assessed across the whole body, but not found for hair length measurements taken from a single location. Group C2 was a homogenous group of similar breed types (quarter horses and thoroughbreds) maintained as a herd at pasture at the same location and exposed to the same ambient temperatures. This explains the shorter hair lengths observed throughout the study period for this group.

### 4.2. Coat Condition

Coat condition changes were assessed in multiple ways in this study: by quantitatively measuring hair length (discussed above) and hair shedding scores; collating responses to BMQs related to bi-monthly owner perceived changes in coat condition; a final questionnaire that assessed response frequencies of reported changes in coat condition clinical signs related to hypertrichosis; and assessment of overall perception of coat condition changes for the entire study. Hair shedding scores were increased in the blue-light-treated group in January and February, indicating an earlier transition of follicles to active shedding of the winter coat in the spring when compared with controls. The stimulatory effect of light on shedding of the hair coat in healthy horses is well supported [15,20,57]. Kooistra and Ginther (1975) found a similar advancement of 35 days in hair shedding in mares maintained under long-day photoperiod treatment from December [57]. Advancement of shedding was also observed in goats maintained under summer lighting and temperature [15]. Similarly, O’Brien et al. (2020) found that both horses and ponies shed earlier when wearing blue light masks activated in late November [20]. It was also evident from the shedding scores that moulting of the summer coat was prolonged in treated horses, as higher scores were again reported in November and December in group T only. This suggests that blue-light-treated horses experienced more pronounced shedding of the summer coat as the winter coat grew in. In the spring, hair follicles in seasonal mammals transition gradually from telogen, through early anagen (when they shed), and into anagen or active growth [61], and this occurs again to a lesser extent as the summer coat is replaced by the growth of the longer winter coat [62]. This finding of more active shedding in the treated group has important implications for horses with PPID, whereby abnormal shedding patterns impact welfare due to difficulties with thermoregulation. The results suggest that use of blue light masks may be a useful treatment option to help minimise the frequent clipping of the coat that is recommended to ensure comfort for these horses [63]. Similar to O’Brien et al. (2020) [20], the results of this study support the use of extended daily blue light administration of 15 h duration as an alternative to clipping to improve shedding in the spring and autumn months for PPID horses. Given the circannual rhythmic nature of the hair growth cycle, it is likely that continuous exposure to the same long-day photoperiod may eventually lead to refractoriness in the hair follicle [18] and the transient growth of a heavier coat. The likelihood of this happening has not been researched to date. However, it is proposed that the benefits of blue light therapy for coat condition and wellness outweigh the occasional need for coat clipping. Furthermore, horses maintained outdoors at pasture during the natural longer days of summer would not need the additional blue light stimulation that a light mask provides.

Increased shedding also implies a reduced hair coat thickness and this is supported by the significantly higher number of participants in group T who reported improved coat condition in their horses in the April BMQ. Notably, this occurred one month after the peak in hair shedding scores of group T in March. A similar trend was seen in group C1, although it occurred later in the year, where 52% of respondents reported that coat condition improved in the June BMQ following the peak in the shedding scores in April. O’Brien et al. (2020) reported coat condition scores which tended toward a shorter, sleeker summer coat earlier in a blue-light-treated group than in a group exposed to natural daylight and this was maintained into autumn in horses receiving extended photoperiod from July [20]. Thus, it is likely that participants in group T similarly perceived the appearance of a summer-like coat in their horses earlier in the year and that this was maintained for longer in response to continued exposure to blue light. Finally, hypertrichosis-related clinical signs were reported as decreased by 71% of group T respondents compared with 6% of group C1 in the final questionnaire. Combined with a significantly greater proportion of group T respondents reporting generally improved coat condition in their horses at the end of the study, these results support the hypothesis that blue light treatment reduces hypertrichosis in horses with PPID.

Previous research has suggested that medication with pergolide mesylate increased shedding in horses with PPID [9]. In our analysis of shedding scores, only 4 of 22 horses in the treatment group and 1 of 19 in the control group were not receiving a daily pergolide dose. While it is not possible to ascertain with certainty that the higher recorded shedding scores were a result of an additive effect of blue light plus pergolide treatment, or an effect of blue light alone, the results highlight that the combined treatments improve shedding in horses with PPID.

### 4.3. Alertness

In the current study there were increased reports of higher energy/alertness in group T horses in February, following two months of wearing the blue light masks. While assessment of energy/alertness may be considered subjective in nature, participants in this study were very engaged in observing and reporting any changes in behaviour of their horses, which is typical of horse owners invested in the well-being of their charges and where they interact with them daily for their care and to administer medication. While the potential for observer bias cannot be ruled out, this finding agrees with a recent controlled study with research-trained observers blinded to treatment, which showed increased wakefulness by day in stabled horses maintained under blue-enriched LED light [64]. The Equilume light masks used in this study deliver light at 465 nm, which is the wavelength found to optimally suppress melatonin and result in concurrent increases in alertness [65]. Indeed, bright light therapy in human subjects has exerted positive effects on alertness after just two weeks of treatment [66], and a recent review confirms the widespread reports of the benefits of blue light for increased human attention, alertness, and reaction time [67]. Furthermore, bright light was shown to rescue circadian behaviour and brain dopamine abnormalities in diurnal rodents exhibiting experimentally induced seasonal affective disorder [68]. Therefore, the finding of increased alertness in blue-light-treated horses early in the study is not surprising. The fact that no significant association was found in the subsequent BMQs may reflect the design of the questions, whereby participants were asked to indicate if they had observed an ‘increase,’ ‘decrease’ or ‘no change’ in the given parameter in the two preceding months since the last questionnaire was completed. It is conceivable that participants may not have reported the same change repeatedly if it was not considered exponential. Therefore, it is not possible to judge from the collection of six BMQs whether the changes in alertness in these horses were sustained throughout the year, unless you assume that reports of ‘no change’ equate to a sustained state of increased energy and alertness from the month of the initial reported increase. Indeed, it is possible that the perceived alertness was sustained and in turn contributed to the perceived reported improvement in quality of life for these animals. As depression and lethargy are common clinical signs of PPID [1], this result suggests a positive impact of blue light therapy for PPID horses.

### 4.4. Fat Coverage

Over 80% of responses to BMQ questions about appetite, urination, drinking, general sociability, and stereotypical behaviours reported “no change.” These parameters were initially included so that the questionnaires encompassed all health parameters known to be affected by PPID. However, to capture the exact frequency of these behaviours would require close monitoring and they are not easily quantifiable without the use of appropriate scales or ethograms and research-trained observers. Other parameters, including coat condition, coat shedding, fat coverage, muscle tone, body condition, and occurrence of infections and other clinical signs, are more easily assessed and recorded. Study participants received detailed video instructions on how to correctly assess BCS by manual palpation of multiple areas on the horse to assess for extent of fat coverage. While there were no reported differences in BCS over time or between groups, a noteworthy finding is the significant reduction in fat coverage reported by group T in both the April and June BMQ. In addition, five participants in group T indicated decreases in regional fat deposits as a clinical sign in the final questionnaire and individual comments submitted included “lost some weight in her hind end and girth,” “loss of fatty deposits along ribs and buttocks,” and “less fatty pads in neck and rump,” which appear to reference localized fat reduction. Abnormal fat accumulation, while considered a clinical sign of PPID, is more commonly associated with the common comorbidity ID [2]. Previously, pergolide was shown to improve supraorbital fat deposits in horses with PPID with a 33% success rate, but there was no effect on reducing fat deposits elsewhere [69]. While the mechanism by which extended photoperiod using blue light reduces abnormal fat deposits remains unknown, potential pathways may involve photoperiod regulation of seasonal changes in IGF-1 [70], or leptin [24], that in turn regulate seasonal weight changes in horses [71]. Based on our results and previous research, a future worthwhile study would be to evaluate the effects of extended photoperiod using blue light on metabolic parameters, including regional fat accumulations, in non-medicated horses with PPID and ID and where nutritional composition of the diet is controlled for.

### 4.5. General Health

There is amassing evidence in the human medical literature of the benefits of blue light exposure by day for optimum functioning of the circadian system and the impact this has on improved health and immunity [22,50]. Blue wavelength light optimally entrains the circadian system by stimulating ipRGC cells in the eye that rapidly communicate the signal first to the master clock in the suprachiasmatic nucleus [21], and then onwards to peripheral clocks located in almost every body tissue [72]. Recently, an LED stable lighting system comprising blue-enriched daytime light was shown to strengthen rhythms of core circadian clock genes in a peripheral tissue in racehorses, where rhythmicity was absent under standard stable lighting [73]. Until now, the impact of blue light on physiological processes regulated by the circadian clock in horses has been limited to reproduction [40,41,42] and hair shedding processes [20,74]). The findings in this study, whereby owners of PPID horses treated with blue light reported a reduction in clinical signs of disease, should encourage further investigations of the impact of lighting that strengthens circadian rhythmicity on health and immunity in horses. One of the most important mediators of the circadian signal in the body, melatonin, is itself a powerful antioxidant [75]. Regulation of melatonin production by blue light is well established in humans [76] and horses [43]. Importantly, it has been reported that exposure to blue light wavelengths during the day caused an increase in peak melatonin during the night in humans [77] and a 7-fold increase in rats [78], highlighting its potential role in mediating the health improvements reported in this study.

Health is a primary influencer of an animal’s quality of life. An important finding of this study was that 50% of participants in the blue-light-treated group reported an improvement in their horses QoL on the final questionnaire. This is despite the advanced age of horses in this study (median age was 23 years) with a progressively debilitating neurodegenerative condition. Recently, there is increasing emphasis placed on early diagnosis and treatment of PPID [14], so investigating the efficacy of blue light treatment in younger horses (~15 years) with a moderate to high pre-test probability of disease will be important to explore.

### 4.6. Study Limitations

Certain limitations are inherent to studies involving participant-owned horses. In addition to variation in management and location, bias in reporting observations must be considered, especially as participants could not be blinded to treatment. Many participants were aware of the potential benefits of blue light for hair coat and hypertrichosis due to availability of previously published research and marketing material related to the Equilume light mask, and this may have affected their perception and responses to questions regarding hair coat. However, no information was provided to participants about the potential effect of blue light on other clinical signs of PPID or health indicators, and they were informed that this information was being collected to assess changes in health parameters associated with the condition. While regular communication with participants was maintained throughout the study, the daily management, charging and maintenance of light masks were outside of the researchers’ control given the wide geographical distribution of study participants. Considerable efforts were made to monitor study progression remotely via a dedicated Facebook page and email correspondence, and frequent reminders were sent to complete monthly and bimonthly tasks. In addition, the electronic submission of monthly date-stamped photographs of each participant’s horse, taken on the day of sampling, helped ensure the reliability of the longitudinal data. The responses to the BMQs and the final questionnaire remain owner-reported and subjective and should be interpreted in this light. Nonetheless, studies of this kind remain informative and an important source of information to guide future research in the absence of dedicated research herds of PPID horses in sufficient numbers.

## 5. Conclusions

Circannual rhythms in hair length changes did not differ between blue-light-treated or control horses with PPID, and no seasonal differences in guard hair length was apparent between groups. Increases in hair shedding and improvements in coat condition and clinical signs associated with hypertrichosis were reported in blue-light-treated horses only and support the hypothesis that extended photoperiod using blue light masks reduces the severity of hypertrichosis in horses with PPID. The reports of reductions in PPID clinical signs and increases in quality of life by over half of the treatment group are promising. While the potential for observer bias means that these findings should be treated cautiously, they do provide justification to further evaluate blue light as a complementary treatment for PPID.

The positive findings reported here from a study cohort of advanced age and PPID stage should prompt further investigation into the potential benefits of blue light for a younger cohort at earlier stages of the condition. Substantial evidence links appropriately timed blue light exposure with strengthened circadian rhythmicity, and the latter with the prevention of disease and improved health in multiple species. This study builds on these links in horses and provides impetus to further study the effects of blue light for horses with PPID in order to better inform management of this condition.

## Figures and Tables

**Table 1:** Study group characteristics for group T (blue light treatment group), group C1 (medicated control group), and group C2 (unmedicated control group). Medication status refers to medicated or unmedicated with pergolide mesylate. ‘Cold blood type’ refers to native and draught breeds and ‘Other’ refers to warmblood and hot blood breeds. Hypertrichosis scores (1–3) were assigned as 1: regional hair coat abnormalities/long hair growth restricted to discrete areas; 2: generalised hair coat abnormalities, slightly to moderately long hair coat; and 3: severely long and/or curly hair coat over the entire body.

Group	Age (Median)	Age (Range)	Sex	Medication Status	Breed Type	Exercise Status	Latitude (°N) (Mean ± SD)	Latitude (°N) (Range)	Hypertr-Ichosis Score
**T** **(*n* = 29)**	23	17–33	Geldings (*n* = 15) Mares (*n* = 14)	Medicated (*n* = 22) Unmedicated (*n* = 7)	Cold blood (*n* = 9) Other (*n* = 20)	Exercised (*n* = 17) Not exercised (*n* = 12)	51.2 ± 5.84	33–61	1: *n* = 7 2: *n* = 18 3: *n* = 4
**C1** **(*n* = 23)**	22	19–32	Geldings (*n* = 13) Mares (*n* = 10)	Medicated (*n* = 22) Unmedicated (*n* = 1)	Cold blood (*n* = 10) Other (*n* = 13)	Exercised (*n* = 10) Not exercised (*n* = 13)	46.0 ± 8.93	27–55	1: *n* = 5 2: *n* = 14 3: *n* = 4
**C2** **(*n* = 17)**	25	18–32	Geldings (*n* = 4) Mares (*n* = 13)	Unmedicated (*n* = 17)	Other (*n* = 17)	Not exercised (*n* = 17)	38	38–38	1: *n* = 7 2: *n* = 6 3: *n* = 4

**Table 2:** Number of horses per group (group T = blue light treatment; group C1 = unmedicated control) exhibiting each clinical sign of PPID (as outlined by the EEG [8]) at the time of recruitment to the study, and the number of respondents per group that selected “Increased” or “Decreased” in relation to each clinical sign in the final questionnaire at the completion of the 13-month study.

	Exhibited at Recruitment		Decreased		Increased	
Clinical Sign	Group T	Group C1	Group T	Group C1	Group T	Group C1
Depressed behaviour	13	12	4	1	0	1
Decreased performance capacity	13	7	1	0	0	1
Muscle atrophy/loss of topline	15	15	4	0	4	3
Laminitis/lameness	12	13	2	3	2	2
Regional fat deposits	13	7	5	2	0	0
Frequent infections	1	4	1	0	0	1
Inappetence	4	5	0	0	0	0
Skin infections/conditions	10	7	2	0	0	1
Excessive urination	13	10	1	1	0	0
Excessive drinking	6	6	0	0	1	0
Abnormal sweating	12	9	4	2	0	1
Stomach ulcers	1	2	0	0	0	0
Supraorbital fat deposits	7	5	1	1	0	0
Regional hypertrichosis	2	4	0	1	0	0
Hypertrichosis	20	15	3	0	0	1
Patchy hair growth	6	4	3	0	1	2
Delayed coat shedding	26	18	7	0	2	2
Loss of seasonal hair coat shedding	17	6	2	0	0	1
High parasite load	0	1	0	0	1	0
Dry eye/corneal ulcers	2	0	1	0	0	0
Infertility	1	0	0	0	0	0
Increased mammary gland secretion	0	3	0	0	0	0
Tendonitis/ Desmitis	1	1	0	0	0	0
Abscesses	3	4	0	0	0	1

## Data Availability

Collated data related to anonymized survey responses are available upon request.

## Supplementary Materials

The following supporting information can be downloaded at https://github.com/EquineResearch/PPID (accessed on 23 July 2025): Call for participants; recruitment questionnaire; sample BMQ; final questionnaire.
